# Predicting Age Groups of Reddit Users Based on Posting Behavior and Metadata: Classification Model Development and Validation

**DOI:** 10.2196/25807

**Published:** 2021-03-16

**Authors:** Robert Chew, Caroline Kery, Laura Baum, Thomas Bukowski, Annice Kim, Mario Navarro

**Affiliations:** 1 Center for Data Science RTI International Research Triangle Park, NC United States; 2 Center for Health Analytics, Media, and Policy RTI International Atlanta, GA United States; 3 Center for Health Analytics, Media, and Policy RTI International Berkeley, CA United States; 4 Center for Health Analytics, Media, and Policy RTI International Research Triangle Park, NC United States; 5 Office of Health Communications and Education Center for Tobacco Products US Food and Drug Administration Silver Spring, MD United States

**Keywords:** Reddit, social media, age, machine learning, classification

## Abstract

**Background:**

Social media are important for monitoring perceptions of public health issues and for educating target audiences about health; however, limited information about the demographics of social media users makes it challenging to identify conversations among target audiences and limits how well social media can be used for public health surveillance and education outreach efforts. Certain social media platforms provide demographic information on followers of a user account, if given, but they are not always disclosed, and researchers have developed machine learning algorithms to predict social media users’ demographic characteristics, mainly for Twitter. To date, there has been limited research on predicting the demographic characteristics of Reddit users.

**Objective:**

We aimed to develop a machine learning algorithm that predicts the age segment of Reddit users, as either adolescents or adults, based on publicly available data.

**Methods:**

This study was conducted between January and September 2020 using publicly available Reddit posts as input data. We manually labeled Reddit users’ age by identifying and reviewing public posts in which Reddit users self-reported their age. We then collected sample posts, comments, and metadata for the labeled user accounts and created variables to capture linguistic patterns, posting behavior, and account details that would distinguish the adolescent age group (aged 13 to 20 years) from the adult age group (aged 21 to 54 years). We split the data into training (n=1660) and test sets (n=415) and performed 5-fold cross validation on the training set to select hyperparameters and perform feature selection. We ran multiple classification algorithms and tested the performance of the models (precision, recall, F1 score) in predicting the age segments of the users in the labeled data. To evaluate associations between each feature and the outcome, we calculated means and confidence intervals and compared the two age groups, with 2-sample t tests, for each transformed model feature.

**Results:**

The gradient boosted trees classifier performed the best, with an F1 score of 0.78. The test set precision and recall scores were 0.79 and 0.89, respectively, for the adolescent group (n=254) and 0.78 and 0.63, respectively, for the adult group (n=161). The most important feature in the model was the number of sentences per comment (permutation score: mean 0.100, SD 0.004). Members of the adolescent age group tended to have created accounts more recently, have higher proportions of submissions and comments in the r/teenagers subreddit, and post more in subreddits with higher subscriber counts than those in the adult group.

**Conclusions:**

We created a Reddit age prediction algorithm with competitive accuracy using publicly available data, suggesting machine learning methods can help public health agencies identify age-related target audiences on Reddit. Our results also suggest that there are characteristics of Reddit users’ posting behavior, linguistic patterns, and account features that distinguish adolescents from adults.

## Introduction

Public health campaigns are a primary means for government agencies and nongovernmental organizations to raise awareness about important health issues affecting their communities. Running effective public health education campaigns requires organizations to tailor messages and provide outreach that resonates with their target audience. For example, the US Food and Drug Administration (FDA) Center for Tobacco Products has developed separate public education campaigns on the health consequences of tobacco products for at-risk teens [1]; lesbian, gay, bisexual, and transgender young adults [2]; multicultural youth [3]; rural male teens [4]; and adult smokers [5]. With increased media consumption and interpersonal interactions occurring online, social media platforms have become important in both engaging target audiences in public education campaigns and in understanding behaviors and perceptions around emerging public health issues across these target audiences. However, it can be challenging to apply results from social media analyses because there is limited information about who these data represent, and thus it is difficult to glean information from populations of interest. In tobacco prevention and control, being able to segment social media posts by age-based audience segments, would help researchers and public health agencies identify emerging issues and changes in behaviors and attitudes to facilitate public health surveillance and educational outreach to these at-risk populations. This would allow researchers to naturalistically observe of their target audience and how they interact with the discussion of tobacco products. This study presents a model developed to classify users of the popular social media site *Reddit* into those more likely to be older or younger than 21 years of age. These age categories mirror the federal minimum age for the purchase of tobacco products in the United States, which was amended from 18 years to 21 years of age [6].

Reddit is a public network of online communities organized around people’s interests. As of 2019, an estimated 11% of US adults use Reddit, with an estimated 22% of US adults aged 18 to 29 years using the platform [7]. Unlike other social media platforms such as Twitter that restrict post length, Reddit was designed for long-form entries, which allows users to provide much greater levels of detail in their posts. In addition, Reddit, similar to traditional forums, allows users to respond to posted material in the form of comment chains. This combination of longer post length and an engaged user base encourages active and nuanced discussions across a myriad of topics relevant to the public health community. In recent years, researchers have used Reddit data to understand emerging public health issues, including cannabis [8-10], opioid [11,12], and alcohol [13,14] use.

Recent studies have used Reddit to investigate underage electronic nicotine delivery systems use. Brett et al [15] performed a content analysis on Reddit posts to identify perceptions among Redditors about underage use of Juul. Zhan et al [16] coded conversations about youth using Juul on the now-banned UnderageJuul subreddit. For studies focused on underage electronic nicotine delivery systems use, the authors used text indicators in posts or the subreddit community’s age as proxies for identifying conversations among youth. However, not everyone posting on subreddits such as UnderageJuul are likely youth, and it is likely that youth are having conversations about vaping in other Reddit communities outside of electronic nicotine delivery systems specific subreddits. For these reasons, a broader approach is needed to identify youth who are discussing electronic nicotine delivery systems across Reddit communities.

One way to identify adolescent conversations about electronic nicotine delivery systems is to examine the characteristics of Reddit users’ posting behavior, look for patterns that distinguish adolescents from adults, and develop an algorithm to predict the age of Reddit users. Predicting latent user demographics on social media is a popular area of research, with researchers developing models to infer categories such as gender [17-19], age [20-22], race and ethnicity [23-25], and political orientation [26-28]. Specifically, latent attribute models trained on Reddit data are becoming increasingly popular, with models for inferring geolocation [29], gender [30], and mental health status [31,32], all recently published.

The objective of this study was to develop an age prediction algorithm to classify Reddit users as adolescents or adults, based on publicly available data, to build a tool that would allow for more nuanced analyses of tobacco related discussions occurring on Reddit. This would allow comparisons of adult and adolescent discussions. Our work extends the literature by developing a model with age categories that are meaningful for a broad public health audience.

## Methods

### Ethics

This study was conducted between January and September 2020. It was determined that the study did not entail research with human participants as defined by 45 CFR 46 by the RTI International review board (STUDY00021174) and the FDA Research Involving Human Subjects Committee (2020-CTP-129).

### Identifying Self-Reporting of Age on Reddit

Developing supervised classification models requires ground truth observations labeled with the outcome of interest. To obtain the ages of Reddit users, we used a social media data platform (Brandwatch) to identify publicly available Reddit posts or comments in which users self-reported their age. Brandwatch is a secure cloud computing platform accessible only to registered users, and data that are extracted contain no personal information beyond the publicly posted username. Extracted data were maintained on secure internal servers and no attempts were made to link usernames to individuals. We searched for English language Reddit posts with the phrase “I’m <age>” that were posted between December 1 and December 31, 2019. In the literature, similar approaches have been used to construct large Reddit data sets containing self-reported demographics [33-35]. We were interested in youth (13-17 years), young adult (18-20 years), and adult (21-54 years) age segments. For the youth segment, the search query was “I’m 13” OR “I’m 14” OR “I’m 15,” OR “I’m 16” OR “I’m 17.” A similar strategy was used to identify posts for the young adult (18-20 years) and adult (21-54 years) age categories. The upper age range was chosen to correspond with the target audience’s age range for FDA campaigns, and age range for young and middle age ranges were chosen to reflect the previous (18 years) and current (21 years) minimum age for sale of tobacco products in the United States. The query pool included both original submissions and comments on posts to maximize the likelihood of identifying users reporting their age.

We reviewed the search results and employed multiple data cleaning steps to prepare the datafile for manual coding. First, we manually reviewed the posts pulled and found that some used “I’m <number>” phrases that did not refer to age such as “I’m 50% done” or “I’m 5ft tall.” To exclude these posts, we dropped all posts where we could not find the phrase “I’m <number>” followed by a space or punctuation. Second, posts from subreddits labeled on Reddit’s list of subreddits as “not suitable for work” or “not suitable for life” were excluded to protect coders from sensitive or harmful content. Finally, if a user posted multiple times during the time period observed, we only included the most recent post.

### Manual Labeling of Age from Reddit Posts

Posts for each age range were imported into the SMART annotation app [36] for coding. We trained 3 coders on coding measures and how to use the SMART platform. All coders coded the same test sample of 30 posts or comments across all age groups. There was high interrater reliability across the coders: 82% unadjusted percent agreement and Cohen κ=0.867. Based on the level of agreement for this sample of 30 posts or comments, the full coding was conducted independently.

Coders manually reviewed and labeled self-reported age in posts until they reached a minimum of 625 cases within each of the target age categories. We targeted 625 labeled cases so that we could have at least 500 labeled cases per age category to train and cross validate the algorithm (80%) and 125 labeled cases per age category for a final test set (20%). In Table 1, we report the final number of posts coded for age, as well as the number of posts excluded because they were not relevant or age could not be determined.

**Table 1:** Number of manually labeled Reddit posts by age category (December 2019).

Category			Coded posts or comments, n
**All ages**			2156
	13-17 years		683
	18-20 years		642
	21-54 years		831
	**Excluded^a^**		
		Cannot determine age	252
		Not relevant	154

^a^We excluded posts if age could not be determined (eg, number provided not related to age; multiple ages provided; different language) or if the post was not relevant (eg, user explicitly states they are based outside of the United States; from throwaway accounts).

Originally, we planned on modeling 3 separate age categories (13-17 years, 18-20 years, and 21-54 years), collecting a nearly balanced labeled data set of observations across the 3 ranges of interest; however, after repeated experimentation with the 3-age category model, correctly categorizing members of the middle range (18-20 years) continued to be difficult, and members were often mischaracterized as belonging to either the young or older age range. Given the poor performance for this age range and the recent increase in the US federal age threshold (from 18 years to 21 years), under which the sale of tobacco products, we collapsed the young (13-17 years) and middle (18-20 years) categories into a single category (13-20 years). The implications of this decision are included in the Discussion.

### Collecting User-, Submission-, and Comment-Level Metadata for Manually Labeled Reddit User Accounts

Once the final set of Reddit user accounts were labeled for each age range, we collected publicly available user-, submission- (originating post), and comment-level metadata using Reddit’s application programming interface (API). An API is an interface that receives requests for data or services from outside programs and sends back replies. Reddit’s API allows for registered apps to have limited access to Reddit metadata.

For each Reddit user, we collected their user-level metadata, which included properties of their account, such as the year it was created and their status on Reddit. No personally identifiable information, such as the user’s demographic information or their location, is available through the Reddit API. Some Reddit users had deleted their accounts since we had first identified their age-related post; therefore, we were unable to collect their information. For Reddit users with active accounts, we collected the metadata fields for up to 100 of their most recent submissions and up to 100 of their most recent comments. In cases where the Reddit user did not have 100 submissions or comments, we collected as many as were available. Submissions and comments prior to 2019 were omitted from analyses to prevent the influence of significantly older posts that may have been posted when a user was in a younger age category, and 85 accounts were excluded from the analysis because they were made private or deleted between the initial collection of self-reported age posts and the API calls for collecting user information.

The Reddit API provides detailed metadata fields for each user profile, submission, and comment. The Reddit API returned 50 metadata fields for each user profile, 12,855 metadata fields for each submission, and 322 metadata fields for each comment for a total of 13,227 metadata fields per account. Although the Reddit API returns many data fields, not all were useful for training the algorithm. First, we excluded metadata fields that had 95% or greater missing data (n=7888) or contained no variation (n=5180). Second, we excluded metadata fields that were related to page cosmetics such as text color or video width (n=28). Third, we excluded several complex data types with high cardinality (ie, very uncommon or unique), such as images, HTML, and URLs (n=44).

### Variables for Age Prediction

The fields that we examined for predicting age of Reddit users included summary statistics of metadata fields from the API and other derived variables that may help distinguish adolescent from older age groups (eg, subreddit frequencies, emoji use, reading level of text). In total, we moved into the modeling phase with 1523 variables. Table 2 provides a summary of the variable groups created, as well as counts and examples from each group.

**Table 2:** Variables for modeling age of Reddit users in the training set derived from the comment, post, and user data collected for users whose ages were confirmed by manual labeling.

Variable group	Metadata used	Example	Variables (N=1523), n
Summary statistics	All	Median post score	189
Subreddit frequencies	Posts and comments	Frequency user posted to “Teenagers” subreddit	624
Emoji frequencies	Comments	Frequency of “ 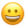 ” out of emojis used by user	101
Literary characteristics	Posts and comments	Average Flesch Reading Ease score	28
Patterns in Posting	Posts and comments	Percentage of user’s posts that were videos	42
Term usage	Comments	TF-IDF^a^ score for the term “school”	539^b^

^a^TF-IDF: term frequency–inverse document frequency.

^b^The number of TF-IDF terms varied across the cross-validation folds based on the comments and submissions vocabulary present in the training portion of each fold. The value presented here is the number of TF-IDF features when calculated on text from the full training set.

### Summary Statistics of Metadata Fields From Reddit API

The first set of variables derived to predict age were constructed directly from the numeric and categorical metadata fields. Fields reporting on user-level attributes were used directly, whereas fields for submissions or comments were derived from aggregations of submissions or comments for each user. For each numeric comment or submission field such as “ups” (the number of upvotes for a post), we derived variables for summarizing their mean, standard deviation, median, first quartile, and third quartile. For binary fields, we calculated the proportion of comments or submissions for which the field’s value was 1. This resulted in 189 summary statistics variables.

### Variables Derived to Help Distinguish Age Groups

In addition to using fields directly available from the Reddit API, we derived further variables to help distinguish between age groups, such as subreddit frequencies, emoji frequencies, literary characteristics, posting patterns, and term usage in comments. In total, there were 624 derived subreddit frequency variables, 101 emoji frequency variables, 28 literary characteristic variables (14 for comment text, 14 for submission text), 42 posting pattern variables, and 539 term usage variables.

Given the importance of text features in previous age classification modeling for social media [37], more detailed information is provided for these sets of variables. For the first set, we turned to established research on Facebook posts by the World Well-Being Project (WWBP) at the University of Pennsylvania. The WWBP [38] had previously identified terms that were used more often by people in 1 of 4 age ranges (13-18 years, 19-22 years, 23-29 years, >30 years). To incorporate this information, each Reddit comment was split into unique words (unigrams), adjacent words (bigrams), and adjacent-word triplets (trigrams). A variable was created for each of the WWBP age group term lists by calculating how many times a term from the list was present in a user’s comment, divided by the total number of n-grams in that user’s comments.

The second set of fields used the term frequency–inverse document frequency (TF-IDF) text representation, which gives a higher weight to terms used frequently within a text document that are also used infrequently across documents. To build these variables, we first cleaned the comment text of English stop words, including many prepositions, conjunctions, and pronouns. Although essential for grammatical English, these terms typically contribute less when there is a need to distinguish between differences in language usage across groups of interest. We then lemmatized each word, reducing conjugated words to their stems (eg, the lemma of *running* is *run*) to better recognize repeated usage of terms across conjugations. Finally, we dropped all numbers from the comment text to avoid age self-report terms that are more likely to occur in our sample than the broader Reddit population. From this processed text, we calculated the TF-IDF score for each unigram, bigram, or trigram between 0.25% and 90% of users in their comments. TF-IDF values were derived only from the data being used for training, whether independently for each cross-validation fold on the training set for hyperparameter tuning and model selection, or on the full training set for a final hold-out comparison to the test set. This resulted in approximately 539 variables when applied to the full training set.

### Model Training

To assess the validity of our findings, we split the data into training and test sets using an 80/20 split. The samples are stratified by age group to ensure similar proportions of age groups across the training and test sets. We performed 5-fold cross validation on the training set to select appropriate hyperparameters and perform model selection to prevent overfitting on the test data.

### Model Evaluation Metrics

To evaluate the model performance, we used area under the receiver operating characteristic curve (AUROC), precision, recall, and F1 score (Multimedia Appendix 1) to assess different aspects of the predictions. All metrics are bounded between 0 and 1, and with all else equal, a higher value indicates a better performing model.

### Model Selection

We assessed several classification algorithms with different underlying assumptions to model age. Specifically, we ran variants of the following methods using the sklearn [39] machine learning framework in Python (Python 3.7): logistic regression with regularization [40], support vector machine [41], *k*-nearest neighbor [42], random forest [43], and gradient boosted trees [44]. Testing several models is motivated by a version of the No Free Lunch Theorem [45], which states that there are no theoretical guarantees that any one machine learning algorithm will work best on all tasks, implicitly promoting an empirical approach to model selection for supervised learning.

### Feature Selection

In addition to developing a model with strong predictive performance, we also aimed to create a model that is interpretable and makes errors easier to diagnose. To help develop a tractable model that mitigates the influence of noisy features, we performed all relevant feature selection [46] using the Boruta method [47] in Python. All relevant feature selection aims to find all features connected to classification decision, as opposed to the minimal optimal selection problem that aims to provide a compact set of features that mimics the optimal classifier. For our implementation, we used 100 iterations and α=.05.

### Feature Importance

To better understand which features contributed to the model predictions, we calculated permutation feature importance scores [43,48] for each feature in the model. The permutation feature importance is the increase in the model’s prediction error after permuting the feature, holding all other features constant. Randomly shuffling the values of the feature effectively breaks the association between the permutated feature, other features, and the outcome. A higher score indicates that the model highly relies on that feature to make accurate predictions, since modifying that feature corresponds to a large drop in model performance. One caution when interpreting permutation importance scores is that the reported contribution from individual features may not be accurate if the features are strongly correlated. We constructed a feature correlation matrix to check this condition and did not find high multicollinearity between any of the remaining features (Figure S1 in Multimedia Appendix 2). Permutations for each feature were repeated 5 times, and the mean and standard deviation were reported.

Although permutation feature importance can help us understand which variables contribute most, it does not provide information on how the features vary across age groups. As a final assessment, we calculated the means, confidence intervals, and 2-sample *t* test comparisons for each transformed feature in the final model. The features were transformed prior to calculating means, intervals, and hypothesis testing because visual inspection of the distributions indicated that the normality assumption for the 2-sample *t* test would not be satisfied for a majority of the covariates. A quantile transformation was applied to each feature to make the sample more closely resemble a Gaussian normal distribution. This univariate transformation first estimates the cumulative distribution function of a variable to a uniform distribution and then maps the values to the desired output distribution (ie, normal distribution) using the associated quantile function.

## Results

### Model Selection

Table 3 summarizes the training set cross-validation model results for each classification algorithm. The reported results use each algorithm’s best performing hyperparameter values discovered during hyperparameter tuning. The model with the highest overall F1 score is the gradient boosted tree (mean 0.77, SD 0.03), followed by random forest (mean 0.75, SD 0.03), and support vector machines (mean 0.74, SD 0.04). The *k*-nearest neighbor model overall had the lowest F1 score (mean 0.59, SD 0.04) of the models considered. Gradient boosted trees were selected as the most appropriate algorithm for this modeling task of those assessed based on their overall higher cross-validation mean and comparable standard deviation for F1 scores across models.

**Table 3:** Classifier performance for predicting manually labeled age of Reddit users based on the full derived variable set.

Age group		Precision, mean (SD)	Recall, mean (SD)	F1 score, mean (SD)	AUROC^a^, mean (SD)	Support, n
**Random forest**						
	13-20 years	0.75 (0.04)	0.91 (0.01)	0.82 (0.02)	—^b^	202
	21-54 years	0.79 (0.03)	0.53 (0.04)	0.63 (0.03)	—	128
	Overall	—	—	0.75 (0.03)	0.83 (0.02)	331
***k*-Nearest neighbors**						
	13-20 years	0.65 (0.04)	0.98 (0.01)	0.78 (0.03)	—	202
	21-54 years	0.84 (0.04)	0.18 (0.04)	0.29 (0.05)	—	128
	Overall	—	—	0.59 (0.04)	0.75 (0.03)	331
**Support vector machine**						
	13-20 years	0.75 (0.04)	0.89 (0.03)	0.82 (0.03)	—	202
	21-54 years	0.76 (0.06)	0.54 (0.05)	0.63 (0.04)	—	128
	Overall	—	—	0.74 (0.04)	0.83 (0.02)	331
**Gradient boosted trees**						
	13-20 years	0.79 (0.04)	0.85 (0.02)	0.82 (0.02)	—	202
	21-54 years	0.74 (0.05)	0.64 (0.04)	0.68 (0.03)	—	128
	Overall	—	—	0.77 (0.03)	0.84 (0.02)	331

^a^AUROC: area under the receiver operating characteristics curve.

^b^Not available or not applicable.

### Feature Selection

All relevant feature selection reduced the number of features from 1523 to 15 (Table 4), resulting in a reduced model with comparable predictive performance (F1 score 0.78) to a model with all variables included (F1 score 0.79).

As a final diagnostic check, a weighted version of the reduced model was run to assess the impact of imbalanced sample sizes of the outcome categories introduced by collapsing age groups. The version of the models with and without the weighting adjustment performed comparably, suggesting that the class imbalance did not drastically impact model results (Table S1 in Multimedia Appendix 3).

**Table 4:** Model performance on the test set for predicting age group of Reddit users using gradient boosted trees classifier with all features vs select features.

Age group		Precision	Recall	F1 score	AUROC^a^	Support, n
**Model with all features (1523 features)**						
	13-20 years	0.81	0.87	0.84	—^b^	254
	21-54 years	0.77	0.67	0.72	—	161
	Overall	—	—	0.79	0.87	415
**Reduced model with selected features (15 features)**						
	13-20 years	0.79	0.89	0.84	—	254
	21-54 years	0.78	0.63	0.70	—	161
	Overall	—	—	0.78	0.86	415

^a^AUROC: area under the receiver operating characteristics curve.

^b^Not available or not applicable.

### Feature Importance

**Figure 1:**
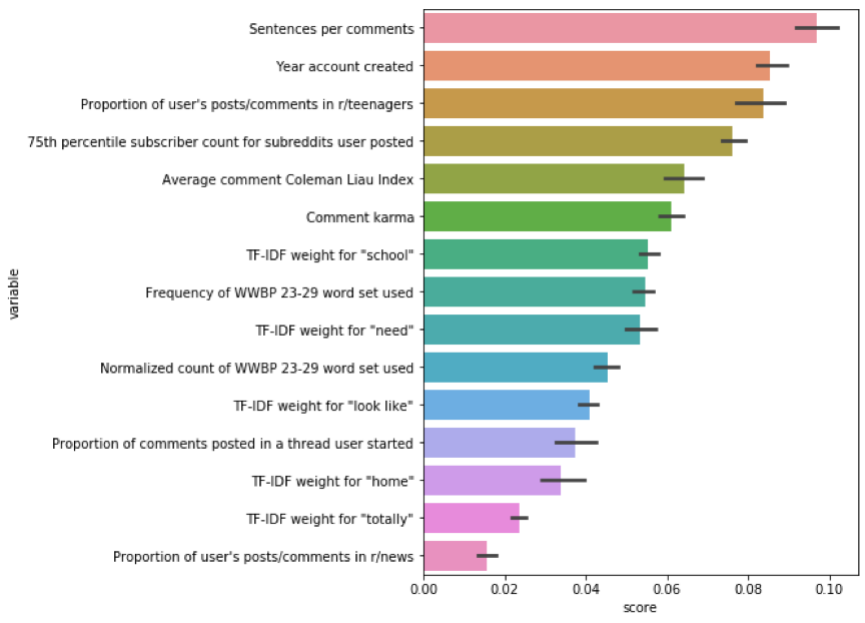
Permutation feature importance scores for top variables in the reduced model for predicting age of Reddit users.

Figure 1 shows the permutation feature importance scores for each variable in the model. Of the feature categories, the largest number (n=7) were derived from terms used in the comment text (TF-IDF weights for “home,” “look like,” “need,” “school,” and “totally”; frequency of WWBP 23-to-29 word set used, normalized count of WWBP 23-to-29 word set used). Three features describe subreddit usage (proportion of comments and posts in *r/teenagers*, proportion of comments and posts in *r/news*, 75th percentile of subscribers for subreddits posted to) and another 3 characterize the user’s literary style when commenting (average Coleman Liau Index for comments, number of sentences per comment, proportion of user comments posted in a thread they started). Only 2 features were derived from user account information that does not require collecting additional posts or comments (account creation year, lifetime comment karma).

The most important model feature was the number of sentences per comment (score: mean 0.100; SD 0.004). The least important feature is the proportion of a user’s posts and comments in the *r/news* subreddit (score: mean 0.017; SD 0.002). Although the text features account for the largest number of relevant variables, features across the categories show high feature importance.

Table 5 summarizes how the most important features varied across age groups. Means for all variables were significantly different, with the exception of the TF-IDF weight for “school,” which exhibited a spike at 0, for which the quantile transformation could not properly account. Table S2 in Multimedia Appendix 4 provides additional nonparametric results for detecting differences in sample distributions (Mann Whitney *U* test and Kolmogorov-Smirnov) that do not compare sample means and are less influenced if the sample transformations fail to meet normality; results from both tests suggest that distributions for the TF-IDF weight for the “school” variable were statistically different (Multimedia Appendix 4) between adolescents and adults in our sample.

For the top variables identified by the feature permutations, when comparing the 13 to 20 years age group, members of the older age group tended to use a larger number of sentences per comment (21-54 years: μ=0.37; 13-20 years: μ=–0.43), higher average comment Coleman Liau Index (21-54 years: μ=0.08; 13-20 years: μ=–0.25), and higher comment karma (21-54 years: μ=0.30; 13-20 years: μ=–0.19). Likewise, when compared with the 21 to 54 years age group, members of the younger 13 to 20 years age group tend to have created an account more recently (21-54 years: μ=1.35; 13-20 years: μ=2.96), have a higher proportion of submissions and comments in the *r/teenagers* subreddit (21-54 years: μ=–5.11; 13-20 years: μ=–3.49), and post more in subreddits with higher subscriber count (21-54 years: μ=0.33; 13-20 years: μ=–0.14).

**Table 5:** Age group comparisons for the top performing variables (by permutation feature importance).

Variable	Type	Age 13-20 years (n=1014), mean^a^ (95% CI)	Age 21-54 years (n=643), mean^a^ (95% CI)	*t* statistic (*df*)	*P* value
Sentences per comments	Literary characteristics	−0.43 (−0.52, −0.35)	0.37 (0.27, 0.46)	−12.31 (1489.89)	<.001
Year account created	Summary statistics	2.96 (2.79, 3.14)	1.35 (1.10, 1.59)	10.65 (1272.76)	<.001
Proportion of user’s posts or comments in *r/ teenagers*	Subreddit frequencies	−3.49 (−3.67, −3.31)	−5.11 (−5.17, −5.05)	16.69 (1208.10)	<.001
75th percentile subscriber count for subreddits user posted	Summary statistics	−0.14 (−0.23, −0.04)	−0.33 (−0.46, −0.20)	2.41 (1276.15)	.02
Average comment Coleman Liau Index	Literary characteristics	−0.25 (−0.34, −0.16)	0.08 (−0.01, 0.17)	−5.06 (1571.31)	<.001
Comment karma	Summary statistics	−0.19 (−0.25, −0.13)	0.30 (0.22, 0.38)	−9.85 (1309.14)	<.001
TF-IDF^b^ weight for “school”	Term usage	−2.46 (−2.65, −2.27)	−2.63 (−2.86, −2.40)	1.14 (1406.18)	.25
Frequency of WWBP^c^ 23-29 word set used	Term usage	−1.23 (−1.39, −1.08)	−0.05 (−0.20, 0.10)	−10.97 (1592.33)	<.001
TF-IDF weight for “need”	Term usage	−1.58 (−1.75, −1.41)	−0.31 (−0.48, −0.15)	−10.48 (1577.27)	<.001
Normalized count of WWBP 23-29 word set used	Term usage	−1.29 (−1.44, −1.14)	0.04 (−0.11, 0.19)	−12.35 (1569.17)	<.001
Proportion of comments posted in a thread user started	Summary statistics	−0.74 (−0.90, −0.57)	−1.41 (−1.62, −1.20)	4.95 (1366.37)	<.001
TF-IDF weight for “look like”	Term usage	−3.04 (−3.22, −2.86)	−1.84 (−2.08, −1.61)	−7.85 (1327.70)	<.001
TF-IDF weight for “home”	Term usage	−3.45 (−3.62, −3.28)	−1.89 (−2.14, −1.65)	−10.24 (1252.67)	<.001
TF-IDF weight for “totally”	Term usage	−4.23 (−4.37, −4.09)	−2.95 (−3.19, −2.71)	−8.92 (1092.36)	<.001
Proportion of user’s posts or comments in *r/news*	Subreddit frequencies	−5.03 (−5.10, −4.97)	−4.29 (−4.48, −4.11)	−7.38 (810.43)	<.001

^a^Quantile transformed means.

^b^TF-IDF: term frequency–inverse document frequency.

^c^World Well-Being Project

## Discussion

### Principal Findings

This study extends the literature on latent user attribute classification on social media by developing a 2-class age classification model for Reddit to better analyze Reddit posts and comments for specific age groups. This classification model can be used to better qualitatively analyze public health issues within the digital space of Reddit. Our results suggest that several different components of a user’s Reddit data help distinguish between users 13 to 20 years and those 21 to 54 years. The relevant text features suggest that the adolescent age group generally use language in their comments that align with expected activities and phrasing often associated with younger age cohorts. For example, the adolescent age group were more likely, on average, to use the term “school” and less likely to use the term “home” than the adult age group. This is perhaps most noticeable in the WWBP 23-to-29 term set variables; both the raw frequency and normalized versions of these variables were found to be relevant and more highly associated with the adult age group.

For subreddits, the adolescent age group on average posted more frequently in *r/teenager* and less frequently in *r/news* than the older age group. They also tend to post in subreddits with more subscribers when compared with the older age group. This may be an indication that popular subreddits are popular because they contain many adolescent users, given that the general Reddit user base is skewed young [49]. The adolescent age group tended to have shorter comments than the adult age group. Younger users also tend to have a higher proportion of comments posted in a thread they started. In terms of account characteristics, the adolescent age group tended to have user accounts that were created more recently and also had lower comment karma. This relationship corroborates the intuition that younger users, because of their age, have had less opportunity to accumulate a long Reddit tenure and comment karma than older users.

All feature categories had at least one variable represented in the final model, except for “emoji” and “patterns in posting.” Although we expected that Reddit users of different age groups would prefer to use emojis more tightly tied to their current life stage (eg, higher usage of “school” or “backpack” emoji for younger users), our findings did not support this conclusion. Previous research has found that the number of emojis posted on public Facebook status updates decreases with user’s age [50,51]. Additionally, web survey results on emoji use also supports the connection between emoji usage and age, with younger participants using both emoji and emoticons more frequently, having more positive attitudes, and having more motives for their usage than their older counterparts [52]. Likewise, although previous studies have identified certain user posting patterns as being associated with age, none of our variables from this group were found to be relevant. For example, Rosenthal and McKeown [53] found variable sets composed of lexical stylistic features (eg, the number of URL and image links) to be predictive of age groups on LiveJournal. Although most studies predicting age of social media users agree that text features are an important signal, future research could help assess which other types of features tend to be more resilient across social media platforms.

Our findings complement those in literature of age prediction on social media and extends the research findings available for Reddit. De Pril [54] analyzed 2 subreddit communities where users routinely self-report age for relationship advice (*r/relationship_advice*) and to meet other Redditors (*r/r4r*) and examined patterns of users posting behavior to predict 2 age categories (<24 years or >24 years for *r/r4r*; <26 years or >26 years for *r/relationship_advice*) using an ensemble of a multilayer perceptron and a *k*-nearest neighbor classifier, reporting an accuracy of 0.77 and AUROC of 0.86 for *r/r4r* and an accuracy of 0.72 and AUROC of 0.79 for *r/relationship_advice*. Gjurkovic et al [34] modeled age on Reddit using linear regression with TF-IDF terms, reporting a Pearson *r*=0.75 between the labeled and predicted ages. Tigunova et al [35] developed a series of age classification benchmark models for Reddit, reporting a hidden attribute model consisting of a convolutional neural network with attention performing best (AUROC 0.88 across 4 age categories—13-23 years, 24-45 years, 46-65 years, and 66-100 years), suggesting that deep learning models trained on text from user’s posts can improve over a bag-of-words approach when predictive performance is more important than interpretation. Although our findings agree with those of previous literature [34,35,54] on Reddit that language features are effective predictors of age, our study suggests that other relevant metadata features, such as subreddit usage, account metadata, and stylistic linguistic features also provide insight into inferring age of Reddit users. Additionally, although difficult to directly compare given differences in the sample composition and age category definitions, our test set (F1 score 0.78) and (AUROC 0.86) perform comparably to previous Reddit age classifications (accuracy 0.72-0.77; AUROC 0.79-0.88 [35,54]).

Our study has several limitations that should be taken into consideration when interpreting the results. First, due to the poor accuracy in predicting the middle age group, we combined the range with that of the lowest age group; therefore, the young group had nearly twice as many users as the older age group (21-54 years). Although the classes were not severely imbalanced, imbalanced class ratios can impact model performance if they provide too great of an incentive to favor the majority class. We ran a weighted version of the final reduced gradient boosted tree model to understand if a weighting correction would impact performance. The results (Table S1 in Multimedia Appendix 3) confirm that performance is nearly the same with or without weighting for the imbalance. Second, users who self-report age may be systematically different than those who do not report age, which may limit generalizability to all Reddit users. Selection biases stemming from what users choose to post or disclose on social media has been documented on Twitter, with significant differences found between the populations of users who do or do not geo-tag their tweets [55,56] and enable or disable location services [56]. Third, due to the evolving nature of language and platform use on social media, studies of this nature need to be continually updated. Previous research has found that language models used for predicting age and gender on social media tend to degrade over time if not retrained, with larger differences (ie, faster changing language) for younger social media users than older social media users [57]. In a production setting, building a model on training data that is nonrepresentative of new examples, whether due to sample selection biases or changes in the environment over time that change the joint distribution of outcomes and covariates, can severely erode the quality of predictions [58]. Monitoring differences in covariates between the training data and new observations is important for determining when to retrain or modify an existing model. Finally, our work used posts of self-reported ages in English. Many important model features outlined in this work likely will not extend to non-English speaking users (eg, language features), and caution should be used if applying this model to accounts of non-English speaking users.

Areas for future research include updating the algorithm to account for changes in linguistic patterns and social media use, examining how users who self-report age may be systematically different than those who do not report age, and considering how algorithms may be applied outside of their intended use case. For example, it is possible that an age prediction algorithm could be applied to target tobacco product advertising to adolescents. Although we believe this is unlikely, given that social media companies including Reddit have policies prohibiting advertising of tobacco products on their platforms that they monitor and enforce [59,60], researchers should consider tradeoffs when developing algorithms including how algorithms may be applied outside of their intended use case.

### Conclusion

Social media platforms are important channels for public health organizations to communicate, and learn about, behaviors and perceptions on emerging health risks such as tobacco and electronic nicotine delivery systems. In particular, being able to identify members of subgroups on social media is essential for effectively promoting behavior change through that medium, as well as for learning about the issues uniquely impacting each target audience. This study presents an age classification model for Reddit users, an important but understudied social media platform in the latent user attribute modeling literature. This model is both interpretable and has competitive predictive performance when compared with previous Reddit age classification results in the literature. Given the persistent adoption of social media usage especially for teens and young adults, any means of helping to reach this audience is useful for improved digital public health campaigns and formative research insights about adolescent vaping behaviors and perceptions.

## Appendix

Multimedia Appendix 1 Definitions of receiver operating characteristic curve (AUROC), precision, recall, and F1 score.

Multimedia Appendix 2 Feature correlation matrix.

Multimedia Appendix 3 Class-weighted gradient boosted tree on the reduced variable set.

Multimedia Appendix 4 Mann-Whitney U test and Kolmogorov-Smirnov test results comparing each model feature by age group.

## Abbreviations

API: application programming interface
AUROC: area under the receiver operating characteristic curve
FDA: Food and Drug Administration
TF-IDF: term frequency–inverse document frequency
WWBP: World Well-Being Project
