# Correction: Predicting Age Groups of Reddit Users Based on Posting Behavior and Metadata: Classification Model Development and Validation

**DOI:** 10.2196/30017

**Published:** 2021-04-30

**Authors:** Robert Chew, Caroline Kery, Laura Baum, Thomas Bukowski, Annice Kim, Mario Navarro

**Affiliations:** 1 Center for Data Science RTI International Research Triangle Park, NC United States; 2 Center for Health Analytics, Media, and Policy RTI International Atlanta, GA United States; 3 Center for Health Analytics, Media, and Policy RTI International Berkeley, CA United States; 4 Center for Health Analytics, Media, and Policy RTI International Research Triangle Park, NC United States; 5 Office of Health Communications and Education Center for Tobacco Products US Food and Drug Administration Silver Spring, MD United States

In “Predicting Age Groups of Reddit Users Based on Posting Behavior and Metadata: Classification Model Development and Validation” (JMIR Public Health Surveill 2021;7(3):e25807) the authors noted one error.

In the originally published manuscript, one sentence under the “Principal Findings” section was incorrect. The following sentence appeared in the second paragraph of this section:

The adult age group tended to have shorter comments than the adult age group.

In the corrected version of the manuscript, this sentence has been changed to:

The adolescent age group tended to have shorter comments than the adult age group.

The correction will appear in the online version of the paper on the JMIR Publications website on April 30, 2021, together with the publication of this correction notice. Because this was made after submission to PubMed, PubMed Central, and other full-text repositories, the corrected article has also been resubmitted to those repositories.

