# Supplementary material for: Predicting Age Groups of Reddit Users Based on Posting Behavior and Metadata: Classification Model Development and Validation
Source: JMIR Public Health Surveill. 2021 Mar 16;7(3):e25807. doi: 10.2196/25807 (PMC8087286; doi:10.2196/25807)
Supplement: Multimedia Appendix 1 [file publichealth_v7i3e25807_app1.docx]

Area under the receiver operating characteristic curve:

The receiver operating characteristic curve is a plot of the true positive rate against the false positive rate at various thresholds. The AUROC, representing the total area under this curve, is equal to the probability that a classifier will rank a randomly chosen instance of Y=1 (adult user) higher than a randomly chosen instance of Y=0 (youth user).

Precision:

The fraction of true positives out of all observations that are predicted to be positive

$$\frac{\boldsymbol{True} \boldsymbol{Positive}}{(\boldsymbol{True} \boldsymbol{Positive}+\boldsymbol{False} \boldsymbol{Positive})}$$

Recall:

The fraction of true positives detected out of all positive labeled examples

$$\frac{\boldsymbol{True} \boldsymbol{Positive}}{(\boldsymbol{True} \boldsymbol{Positive}+\boldsymbol{False} \boldsymbol{Negative})}$$

F1 score:

The harmonic mean of precision and recall. This summary metrics balances the need to find positive examples with the need to reduce the number of false positives.

$$\frac{\mathbf{2}}{\frac{\mathbf{1}}{\boldsymbol{recall}}+ \frac{\mathbf{1}}{\boldsymbol{precision}}}$$
