# Supplementary figures and images for: Predicting Age Groups of Reddit Users Based on Posting Behavior and Metadata: Classification Model Development and Validation
Source: JMIR Public Health Surveill. 2021 Mar 16;7(3):e25807. doi: 10.2196/25807 (PMC8087286; doi:10.2196/25807)

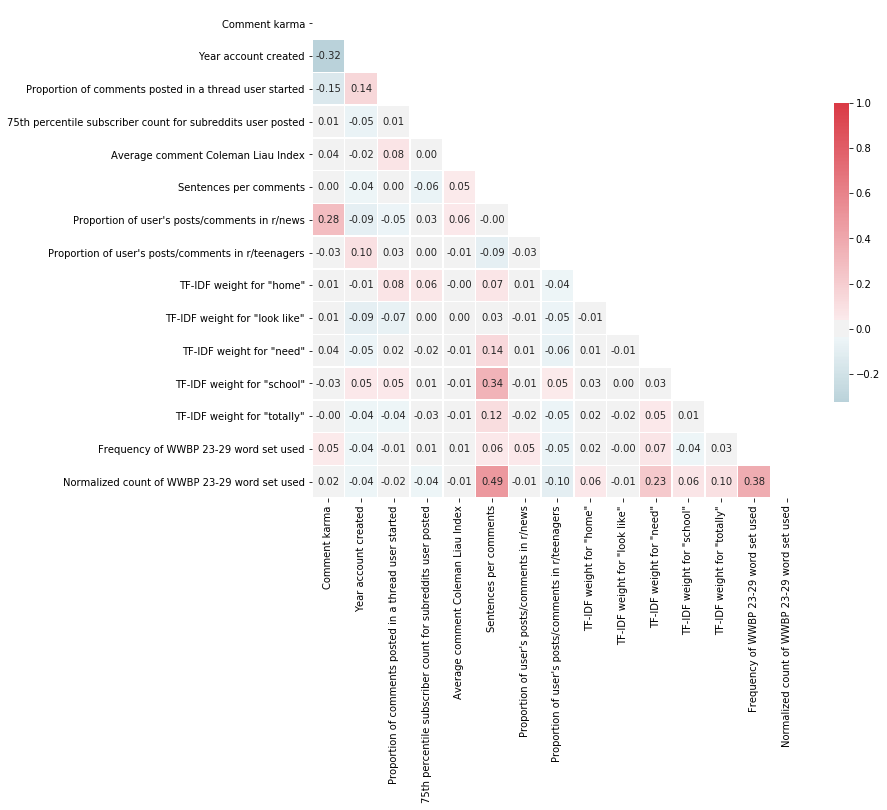

Supplement: Multimedia Appendix 2 [file publichealth_v7i3e25807_app2.docx]
