# Supplementary material for: Predicting Age Groups of Reddit Users Based on Posting Behavior and Metadata: Classification Model Development and Validation
Source: JMIR Public Health Surveill. 2021 Mar 16;7(3):e25807. doi: 10.2196/25807 (PMC8087286; doi:10.2196/25807)
Supplement: Multimedia Appendix 3 [file publichealth_v7i3e25807_app3.docx]

| Age group | Precision | Recall | F1 | AUROC^a^ | Support |
| --- | --- | --- | --- | --- | --- |
| Gradient Boosted Trees |  |  |  |  |  |
| 13–17 | 0.84 | 0.80 | 0.82 | -^b^ | 254 |
| 21–54 | 0.71 | 0.76 | 0.74 | - | 161 |
| Overall |  |  | 0.78 | 0.86 | 415 |

^a^AUROC: area under the receiver operating characteristics curve.

^b^Not available or not applicable.
