# Supplementary material for: Predicting Age Groups of Reddit Users Based on Posting Behavior and Metadata: Classification Model Development and Validation
Source: JMIR Public Health Surveill. 2021 Mar 16;7(3):e25807. doi: 10.2196/25807 (PMC8087286; doi:10.2196/25807)
Supplement: Multimedia Appendix 4 [file publichealth_v7i3e25807_app4.docx]

| Variable | Type | Mann Whitney  U-test | | Kolmogorov-Smirnov Test | |
| --- | --- | --- | --- | --- | --- |
|  |  | statistic | p-value | statistic | p-value |
| Sentences per comments | Literary style | 180,060 | 0.000 | 0.37 | 0.000 |
| Year account created | Profile metadata | 216,818 | 0.000 | 0.29 | 0.000 |
| Proportion of user’s posts/comments in r/teenagers | Subreddit | 246,930 | 0.000 | 0.24 | 0.000 |
| 75th percentile subscriber count for subreddits user posted | Subreddit | 288,241 | 0.000 | 0.12 | 0.000 |
| Average comment Coleman Liau Index | Literary style | 272,328 | 0.000 | 0.17 | 0.000 |
| Comment karma | Profile metadata | 239,939 | 0.000 | 0.18 | 0.000 |
| TF-IDF weight for “school” | Comment text | 308,427 | 0.022 | 0.08 | 0.013 |
| Frequency of WWBP 23–29 word set used | Comment text | 204,373 | 0.000 | 0.32 | 0.000 |
| TF-IDF weight for “need” | Comment text | 230,200 | 0.000 | 0.26 | 0.000 |
| Normalized count of WWBP 23–29 word set used | Comment text | 176,827 | 0.000 | 0.39 | 0.000 |
| Proportion of comments posted in a thread user started | Literary style | 271,161 | 0.000 | 0.14 | 0.000 |
| TF-IDF weight for “look like” | Comment text | 265,301 | 0.000 | 0.21 | 0.000 |
| TF-IDF weight for “home” | Comment text | 241,800 | 0.000 | 0.25 | 0.000 |
| TF-IDF weight for “totally” | Comment text | 261,943 | 0.000 | 0.20 | 0.000 |
| Proportion of user’s posts/comments in r/news | Subreddit | 292,179 | 0.000 | 0.10 | 0.000 |
